# Factors associated with surgical site infection in myocardial revascularization: a retrospective longitudinal study

**DOI:** 10.1590/0034-7167-2023-0108

**Published:** 2023-12-08

**Authors:** Amanda Gubert Pereira, Juliana Martins Lopes, Lorena Cristina Silva Pereira, Aline Guarato da Cunha Bragato, Suely Amorim de Araújo, Valéria Nasser Figueiredo, Vanderlei José Haas, Maria Beatriz Guimarães Raponi

**Affiliations:** I Universidade Federal de Uberlândia. Uberlândia, Minas Gerais, Brazil; II Universidade Federal do Triângulo Mineiro. Uberaba, Minas Gerais, Brazil

**Keywords:** Cardiac Surgical Procedures, Surgical Wound Infection, Infections, Patient Safety, Incidence, Procedimientos Quirúrgicos Cardíacos, Infección de la Herida Quirúrgica, Infecciones, Seguridad del Paciente, Incidencia, Procedimentos Cirúrgicos Cardíacos, Infecção da Ferida Cirúrgica, Infecções, Segurança do Paciente, Incidência

## Abstract

**Objectives:**

to analyze the influence of sociodemographic and clinical variables, as well as the surgical checklist adherence score, on the occurrence of surgical site infection among patients undergoing myocardial revascularization.

**Methods:**

an observational, longitudinal, retrospective study was conducted at a university hospital, involving 266 medical records of patients who underwent myocardial revascularization surgery. Instruments containing sociodemographic, clinical, and infection-related variables were used, along with the Perioperative Surgical Safety Checklist. Descriptive, bivariate, and logistic regression analyses were employed.

**Results:**

surgical site infection occurred in 89 (33.5%) patients. There was a statistically significant association between body temperature outside the range of 36 degrees Celsius to 36.5 degrees Celsius (p=0.01), the presence of invasive devices (p=0.05), surgical procedures with the anticipation of critical events (p<0.001), and the occurrence of infection.

**Conclusions:**

body temperature, the presence of invasive devices, and surgical procedures with the anticipation of critical events were significant factors contributing to an increased risk of infection.

## RESUMO

**Objetivos:**

analisar a influência de variáveis sociodemográficas e clínicas e do escore de adesão ao *checklist* cirúrgico sobre a ocorrência de infecção de sítio cirúrgico entre pacientes submetidos à revascularização miocárdica.

**Métodos:**

estudo observacional, longitudinal, retrospectivo, realizado em hospital universitário, com 266 prontuários de pacientes submetidos à cirurgia de revascularização miocárdica. Utilizaram-se instrumentos contendo variáveis sociodemográficas, clínicas e relacionadas à incidência de infecções de sítio cirúrgico; e a Lista de Verificação de Segurança Cirúrgica Perioperatória. Empregaram-se análises descritivas, bivariadas e regressão logística.

**Resultados:**

a infecção de sítio cirúrgico ocorreu em 89 (33,5%) pacientes. Houve associação estatisticamente significativa entre temperatura fora da faixa entre 36°C e 36,5°C (p=0,01), presença de dispositivos invasivos (p=0,05) e procedimentos cirúrgicos com previsão de eventos críticos (p<0,001) e ocorrência de infecção.

**Conclusões:**

temperatura corporal, presença de dispositivos invasivos e procedimentos cirúrgicos com previsão de eventos críticos foram fatores significativos para o aumento do risco de infecção.

## RESUMEN

**Objetivos:**

analizar la influencia de variables sociodemográficas y clínicas, así como del puntaje de adherencia a la lista de verificación quirúrgica en aparición de infección del sitio quirúrgico en pacientes sometidos a revascularización miocárdica.

**Métodos:**

estudio observacional, longitudinal y retrospectivo realizado en un hospital universitario con 266 expedientes de pacientes sometidos a cirugía de revascularización miocárdica. Se utilizaron instrumentos que contenían variables sociodemográficas, clínicas y relacionadas con la incidencia de infecciones del sitio quirúrgico, así como la Lista de Verificación de Seguridad Quirúrgica Perioperatoria. Se realizaron análisis descriptivos, bivariados y de regresión logística.

**Resultados:**

la infección del sitio quirúrgico se produjo en 89 (33,5%) pacientes. Hubo una asociación estadísticamente significativa entre la temperatura fuera del rango de 36°C a 36,5°C(p=0,01), la presencia de dispositivos invasivos(p=0,05) y los procedimientos quirúrgicos con previsión de eventos críticos(p<0,001) y la aparición de infección.

**Conclusiones:**

la temperatura corporal, presencia de dispositivos invasivos y los procedimientos quirúrgicos con previsión de eventos críticos fueron factores significativos para aumentar el riesgo de infección.

## INTRODUCTION

Surgical site infection (SSI) is the most common healthcare-associated infection (HAI) among patients undergoing surgeries, representing 14-16% of all HAIs^(1)^. A review study highlights that SSIs are considered one of the main risks to patient^(2)^ safety and the third leading cause of infection among hospitalized patients in Brazilian hospitals^(1)^.

Cardiac SSIs are complications that can occur up to 30 days after the surgical procedure^(3)^ and can result in prolonged hospitalization, high hospital and societal costs, and increased patient morbidity and mortality^(1)^. The most common complications of cardiac surgeries are infections. An international study investigating microbial infections after cardiac surgery in cardiac surgery wards, conducted with 123 patients, found that 17-23% of patients develop SSIs after cardiac surgical procedures, as these patients have higher risk factors and vulnerabilities^(4)^.

The occurrence of SSI is based on multiple risk factors associated with the patient’s sociodemographic and clinical profile, the invasive nature of the procedure, the team’s conduct, and the possibility of contamination during surgery^(5)^. Patient-related causes include gender, lifestyle habits, advanced age, nutritional status, and pre-existing conditions^(6)^.

Among the factors associated with the surgical procedure, the complexity of myocardial revascularization, procedure duration, blood transfusion, and the possibility of extracorporeal circulation are noteworthy^(6)^. Regarding the healthcare team, the use of antimicrobial prophylaxis and surgical site antisepsis are important^(7)^.

Finally, there are specific factors related to the risk of surgical contamination, such as hair removal, preparation of the operative area, antimicrobial prophylaxis, and maintenance of the patient’s normal body temperature^(7)^.

The use of surgical safety checklist protocols adopted by hospitals and healthcare professionals, particularly in recent decades, has indicated an improvement in patient safety and a reduction in morbidity and mortality^(8)^, as well as being a potential means to mitigate the occurrence of SSIs.

A study conducted in Dutch cardiac centers aimed to analyze the introduction of a pre-incision checklist on the mortality of 5937 adult patients undergoing cardiac surgery and found that after the implementation of the surgical checklist, the mortality rate within 120 days was significantly lower^(9)^.

The need for evidence-based best practices by the multidisciplinary healthcare team in the context of cardiac interventions and the recognition of potential contributing factors justify the relevance of this study, which can support perioperative nursing care planning in the prevention of surgical site infections by identifying patients at risk of developing them.

## OBJECTIVES

To analyze the influence of sociodemographic and clinical variables, as well as adherence to the surgical checklist score, on the occurrence of surgical site infection among patients undergoing myocardial revascularization.

## METHODS

Ethical Considerations

This study was conducted in accordance with Resolution 466/2012 of the National Health Council and received approval from an Ethics Research Committee. Patient consent was waived due to the use of medical record data available in the hospital’s information system.

### Study Design, Study Site, and Period

This was an observational, longitudinal, retrospective study conducted at a high-complexity university hospital located in the interior of Minas Gerais, Brazil. Data collection took place between July 2021 and February 2022. The Strengthening the Reporting of Observational Studies in Epidemiology (STROBE) checklist for cross-sectional studies was adopted to guide the research^(10)^.

### Sample; Inclusion and Exclusion Criteria

Medical records of patients who underwent myocardial re-vascularization surgery between January 2017 and December 2020, and were 18 years of age or older, were included. Patients who underwent emergency surgery or had a previous infectious focus recorded were excluded. The recruitment process was non-probabilistic.

### Study Protocol

Data collection utilized an instrument developed by the researchers, which included sociodemographic variables (age, gender), clinical variables (pre-existing conditions, lifestyle habits, length of hospitalization, duration of surgery, duration of anesthesia, American Society of Anesthesiologists (ASA) score, type of anesthesia), and variables related to surgical site infection occurrence (presence of SSI, symptoms and time of onset, use of antimicrobial agents, drugs used, duration of use, characteristics of surgical wound contamination, and pathogens present). The Surgical Checklist for Patient Safety and Prevention of Surgical Site Infection^(11)^ was also used. This perioperative surgical safety checklist comprises items covering recommended practices by the World Health Organization that address key measures for infection prevention, divided into five moments: admission to the operating room, before the start of anesthesia and draping, before surgical incision, before leaving the operating room, and before leaving the operating suite^(11)^. From this checklist, the adherence score can be calculated by summing the items, dividing the result by the total number of items, and multiplying by 100%^(11)^.

Patient data regarding sociodemographic and clinical variables and adherence to safety measures were obtained from medical records, to which the researchers had access through the Medical Records Department. Data on postoperative surgical site infection were obtained from the records of the Hospital Infection Control Center (CCIH).

### Results Analysis and Statistics

Data were analyzed using the Statistical Package for the Social Sciences (SPSS) for Windows, version 23. Absolute and percentage frequency distributions were used for categorical variables, and measures of central tendency (mean) and variability (standard deviation) were used for quantitative variables. To identify the influence of sociodemographic and clinical variables on infection, association measures in contingency tables (relative risk, odds ratio, and respective confidence intervals) were used, followed by logistic regression with adjustment for other potentially relevant variables. Inferential analyses considered a significance level of 5% (α=0.05).

## RESULTS

A total of 266 medical records of patients who underwent myocardial revascularization surgery were included in the study. There was a predominance of men (187; 70.3%), with a mean age of 62.73 years (SD=10.08), ranging from 33 to 86 years. It was evident that patients had comorbidities such as diabetes mellitus (135; 50.8%), systemic arterial hypertension (SAH) (139; 52.3%), and obesity (57; 21.4%). Regarding smoking and alcohol consumption, the majority of patients reported alcohol consumption as one of their lifestyle habits (240; 90.2%), while only 13 (4.9%) reported smoking (Table 1). The ASA score was not fully documented due to incomplete records (04; 1.6%).

**Table 1:** Characterization of study participants, Uberlândia, Minas Gerais, Brazil, 2022

Variables	n	%
Sex		
Female	79	29.7
Male	187	70.3
Type of anesthesia		
General	260	97.4
Other	06	2.6
Lifestyle habits		
Alcoholism	240	90.2
Smoking	13	4.9
Does not mention alcoholism or smoking	13	4.9
ASA Score*		
01	03	1.1
02	41	15.4
03	194	72.9
04	23	8.6
05	01	0.4

* Classification of physical status according to the American Society of Anesthesiologists.

The average length of hospitalization was 25.25 days (SD=20.43), with a minimum of three days and a maximum of 106 days. Most patients were classified as ASA 3 (194; 72.9%) regarding physical status and underwent general anesthesia (260; 97.4%) for their surgeries.

Table 2 displays data related to the surgical checklist for patient safety and prevention of surgical site infection at the five moments. The majority of participants underwent preoperative bathing (248; 93.2%) and had informed surgical and anesthetic consent (241; 90.6%). Less than half of the participants had an ideal body temperature (96; 36.1%), and slightly more than half had invasive devices preoperatively (149; 56.0%), with a predominance of peripheral venous access (139; 93.3%).

**Table 2:** Distribution of participants undergoing myocardial revascularization (N=266) according to items present in the Surgical Checklist for Patient Safety and Prevention of Surgical Site Infection, Uberlândia, Minas Gerais, Brazil, 2022

	Yes		No		Not applicable		Omitted	
	n	%	n	%	n	%	n	%
Admission to the surgical center								
Identification bracelet presente	161	60.5	96	36.1	-	-	09	3.4
Preoperative bath	248	93.2	02	0.8	-	-	16	6.0
Use of antibiotics in the last 24 hours	22	8.3	237	89.1	-	-	07	2.6
Is under specific precautions	11	4.1	237	89.1	-	-	18	6.8
Surgical site marked	48	18.0	126	47.4	-	-	92	34.6
Known or declared allergy	30	11.3	232	87.2	-	-	04	1.5
Pre-anesthetic evaluation form present	175	65.8	91	34.2	-	-	-	-
Informed anesthesia consent presente	241	90.6	25	9.4	-	-	-	-
Informed surgical consent presente	241	90.6	25	9.4	-	-	-	-
Hair removal performed	183	68.8	36	13.5	47	17.7	-	-
Patient identification label in the medical record	197	74.1	61	22.9	-	-	08	3.0
Presence of invasive devices	149	56.0	103	38.7	-	-	14	5.3
Body temperature between 36 and 36.5°C	96	36.1	86	32.3	84	31.6
Before the start of anesthesia and the distribution of surgical fields								
Verified patient's name and registration number	182	68.4	05	1.7	-	-	79	29.7
Anesthesia machine tested and operational	193	72.6	-	-	73	27.4
Vital signs monitoring installed and operational	151	56.8	34	12.7	-	-	81	30.5
Difficult airway / risk of bronchoaspiration	39	14.7	116	43.6	19	7.1	92	34.6
Assistive equipment available	74	27.8	62	23.3	-	-	130	48.9
Risk of significant blood loss (>500ml)	190	71.5	03	1.1	-	-	73	27.4
Confirmed blood reserve	197	74.1	-	-	69	25.9
All necessary materials and supplies are present	203	76.3	05	1.9	-	-	58	21.8
Scalpel blade positioned	180	67.7	86	32.3	-	-	-	-
Patient positioned to prevent injuries	125	47.0	91	34.2	-	-	50	18.8
Essential diagnostic images can be viewed	209	78.6	01	0.4	05	1.9	51	19.1
Surgical site antisepsis performed	184	69.2	26	9.8	-	-	56	21.0
Blood glucose level less than 200mg/dL	110	41.4	12	4.5	-	-	144	54.1
Before the surgical incision								
There are critical events foreseen for the surgical procedure	41	15.4	179	67.3	-	-	46	17.3
There are critical events foreseen for the anesthetic procedure	45	16.9	174	65.4	-	-	47	17.7
There are critical events foreseen for the nursing procedure	-	-	222	83.5	-	-	44	16.5
Prophylactic antibiotic administered in the last 60 minutes	176	66.2	79	29.7	11	4.1	-	-
Increased FiO2* in patients with normal lung function undergoing tracheal intubation	112	42.1	86	32.3	-	-	68	25.6
Before leaving the operating room								
The count of compresses and gauzes is correct	163	61.3	12	4.5	45	16.9	46	17.3
The count of instruments and needles is correct	204	76.7	15	5.6	-	-	47	17.7
Material collected (anatomopathological or any other)	12	4.5	226	85.0	-	-	28	10.5
The request is correctly identified	07	2.6	-	-	227	85.3	32	12.1
Were there any issues with materials, equipment, or instruments?	01	0.4	215	80.8	-	-	50	18.8
Does the patient have any skin lesions related to positioning or the operative act?	01	0.4	55	20.7	-	-	210	78.9
Increased FiO2* after extubation.	85	32.0	107	40.2	-	-	74	27.8
Before leaving the surgical center								
Identification bracelet presente	216	81.2	48	18.0	-	-	02	0.8
Presence of invasive devices	208	78.2	27	10.2	-	-	31	11.6
Transoperative and anesthetic record in the medical record	256	96.2	10	3.8	-	-	-	-
Surgical description in the medical record signed	76	28.6	190	71.4	-	-	-	-
Prolongation of surgical antimicrobial prophylaxis in the postoperative period	248	93.2	18	6.8	-	-	-	-

* *FiO2 - Fraction of Inspired Oxygen.*

Before the start of anesthesia and draping, diagnostic images (209; 78.6%) and necessary materials (203; 76.3%) were available for most patients. Before surgical incision, most patients did not have critical events predicted for the surgical (179; 67.3%), anesthetic (174; 65.4%), or nursing (222; 83.5%) procedures. Before leaving the operating room, the correct count of compresses and gauzes (163; 61.3%) and instruments and needles (204; 76.7%) was performed in most procedures. Finally, before leaving the operating suite, there was a predominance of unsigned surgical descriptions in the medical records (190; 71.4%), the presence of invasive devices (208; 78.2%), and the extension of postoperative surgical antimicrobial prophylaxis (248; 93.2%). In addition to item analysis, the total adherence score was calculated, ranging from 11.00% to 72.00%, with a mean of 46.78%, a median of 50.00%, and a standard deviation of 11.80%.

Among the study participants, 89 (33.5%) developed surgical site infection (SSI) after cardiac surgery. The patients had an average time to the onset of infection symptoms of 12.03 days (SD=8.92). Various signs and symptoms were reported by the patients, as described in Table 3. The majority of patients presented with wound discharge (73; 82%).

**Table 3:** Signs and symptoms reported by patients after undergoing myocardial revascularization, Uberlândia, Minas Gerais, Brazil, 2022

Symptoms	Yes		No	
	n	%	n	%
Hyperemia	21	23.6	68	76.4
Secretion in the surgical wound	73	82.0	16	18.0
Fever	17	19.1	72	80.9
Pain	28	31.5	61	68.5
Respiratory insufficiency	08	9.0	81	91.0
Decreased level of consciousness	01	1.1	88	98.9
Swelling	06	6.7	83	93.3
Tachycardia	01	1.1	88	98.9
Nausea and vomiting	01	1.1	88	98.9
Syncope	02	2.2	87	97.8
Dehiscence	05	5.6	84	94.4

The most prevalent microorganisms were Klebsiella pneumoniae (17; 19.3%) and Staphylococcus aureus (16; 18.2%). Of the 89 patients who had SSI, 84 (94.4%) received antimicrobial treatment, with teicoplanin being the most commonly used drug (31; 34.8%), followed by piperacillin combined with tazobactam (26; 29.2%) and cefepime (26; 29.2%). The patients had an average duration of antimicrobial use of 13.56 days (SD=7.10), with a minimum of one day and a maximum of 46 days.

Among the patients who had SSIs, eight (9.1%) progressed to death, with the causes being septic shock (4; 50.0%) and cardiogenic shock (4; 50.0%).

When assessing the association between sociodemographic and clinical variables, adherence score, and the occurrence of surgical site infection in patients undergoing myocardial revascularization, it was evident that body temperature outside the range of 36 to 36.5°C, the presence of invasive devices, and the anticipation of critical events in the surgical procedure presented a higher risk for the development of SSI, with statistically significant differences (Table 4).

**Table 4:** Bivariate analysis and logistic regression considering the occurrence of surgical site infection and sociodemographic and clinical variables of patients undergoing myocardial revascularization surgery, Uberlândia, Minas Gerais, Brazil, 2022

Variables	Presence of infections		RRφ(IC)₸	OR≈(IC)₸	aOR∞(IC)₸	p^8^
	Yes n (%)	No n (%)				
Gender						
Female	32 (40.5)	47 (59.5)	1.32 (0.94 - 1.87)	1.55 (0.89 - 2.68)	0.65 (0.27 - 1.55)	0.33
Male	57 (30.5)	130 (69.5)	ref	ref	ref
Body temperature						
36-36.5ºC	26 (27.1)	70 (72.9)	2.23 (1.20 - 4.14)	1.67 (1.12 - 2.50)	2.80 (1.19 - 6.57)	0.01
Other	39 (45.3)	47 (54.7)	ref	ref	ref
Invasive devices						
Yes	56 (37.6)	93 (62.4)	1.21 (0.84 - 1.72)	1.33 (0.78 - 2.27)	2.35 (1.01 - 5.45)	0.05
No	32 (31.1)	71 (68.9)	ref	ref	ref
Predicted critical events for surgery						
Yes	21 (51.2)	20 (48.8)	1.79 (1.23 - 2.62)	2.65 (1.31 - 5.27)	3.91 (1.42 - 10.73)	<0.001
No	51 (28.5)	128 (71.5)	ref	ref	ref
Surgical site antisepsis performed						
Yes	65 (35.3)	119 (64.7)	0.32 (0.11 – 0.96)	0.24 (0.6 – 0.82)	0.11 (0.01 – 0.99)	0.06
No	03 (11.5)	23 (88.5)	ref	ref	ref
Age	-	-	-	-	1.00 (0.95 - 1.05)	0.87
ASA Score	-	-	-	-	1.20 (0.55 - 2.61)	0.63
Adherence score	-	-	-	-	1.03 (0.97 - 1.10)	0.25
Number of comorbidities	-	-	-	-	1.26 (0.81 - 1.96)	0.29

* Table of regression of variables associated with infection; ϕ RR - Relative risk; ₸ CI - Confidence interval; ≈ OR - Unadjusted odds ratios; ∞ aOR - Adjusted odds ratios; 8level of significance (p <0.05).

The results indicate that patients with body temperatures below 36°C or above 36.5°C are 2.8 times more likely to develop SSI. Having invasive devices increased the chance of post-myocardial revascularization SSI by 2.35 times. Surgical procedures with an anticipation of critical events had a 3.91 times greater chance of SSI occurrence. Despite the initial belief that adherence to the surgical checklist could be a protective factor against SSI occurrence, this study, however, revealed that this variable did not have a statistically significant relationship with the infection.

## DISCUSSION

Patients who participated in a study conducted in a nursing outpatient clinic for post-cardiac surgery wound care, aimed at verifying the clinical-surgical profile and early identification of infection signs, presented a similar profile to the patients in the present study, with a predominance of men, the elderly, and individuals with diabetes mellitus and systemic arterial hypertension^(12)^.

In the current study, low adherence scores to the checklist and the absence of a relationship between adherence to best practices and the occurrence of surgical site infection were evident. A retrospective cohort study conducted at a cardiology institute with the goal of identifying risk factors for SSI in cardiac surgeries also demonstrated that a set of preventive measures, encompassing recommended practices for infection prevention, did not associate with a decrease in SSI risk^(13)^.

However, the literature also presents different findings. A systematic review and meta-analysis of intervention studies analyzed studies from 20 different countries and aimed to assess and quantify the effects of improvement interventions on hospital-acquired infection mortality and surgical site infections in the perioperative environment. Thirty-one studies were included in qualitative synthesis, and 28 were included in the meta-analysis, which demonstrated a 50% reduction in SSI and a 68% reduction in mortality after using the World Health Organization (WHO) Surgical Safety Checklist^(14)^.

Another review conducted in Sub-Saharan Africa systematically reviewed existing literature on hospital quality improvement in surgical and anesthesia procedures. It included 49 articles from MEDLINE, EMBASE, Global Health, CINAHL, Web of Science, and gray literature. The most mentioned interventions were the use of surgical safety checklists (14; 28.6%) and a reduction in SSI after checklist implementation (12; 24.5%). The study highlights the importance of evidence-based interventions for patient safety^(15)^.

A study conducted at seven non-academic Dutch cardiac centers aimed to analyze the introduction of the Isala Safety Check, a specific pre-incision checklist for safe cardiac surgery, on the mortality of an adult population. The research included 5937 patients undergoing cardiac surgery, with 3219 (52%) not using the Isala checklist and 2718 (46%) using it. The surgical infection rate showed a reduction, with 0.7% in the first group and 0.4% in the second, and the mortality within 120 days of analysis was also lower in patients who used the checklist, with 3.0% in the first group and 1.7% in the second^(9)^.

Generally, cardiac surgeries result in high rates of SSI. A study conducted at a highly complex public university hospital specializing in cardiac and thoracic surgeries in São Paulo aimed to describe the profile of patients who developed mediastinitis after cardiac surgery postoperatively, analyzing outcomes related to hospitalization duration, rehospitalization necessity, and instituted antibiotic therapy. The study analyzed 86 medical records and showed that 78 (90.7%) of the patients underwent culture collection, and 68 (79.1%) of them had an infection. The main symptoms of the infection were purulent wound drainage (68; 79.1%) and sternal pain (37; 43%). As pathogens causing the infection, 21 (30.9%) patients had Staphylococcus aureus, and 14 (20.6%) had Klebsiella pneumoniae^(6)^.

Another study was conducted at King Khalid University Hospital in Saudi Arabia and aimed to determine the incidence, risk factors, identify organisms, and evaluate outcomes of surgical wound infections after cardiac surgery. The study included 1241 patients undergoing cardiac surgeries, and 40 (3.2%) of them experienced post-surgical SSI. Among the causative pathogens, the main ones were methicillin-sensitive Staphylococcus aureus (18; 45%), methicillin-resistant Staphylococcus aureus (5; 12.5%), Klebsiella pneumoniae (5; 12.5%), and Pseudomonas aeruginosa (5; 12.5%)^(16)^.

Patients who develop cardiac SSIs have a higher risk of mortality. A retrospective study conducted in general hospitals in Sweden aimed to investigate the association between deep surgical wound infection after cardiac surgery and mortality. It analyzed 114,676 patients undergoing cardiac surgeries, of which 1516 (1.3%) developed post-surgical infections, with the majority (1057; 69.7%) being patients undergoing myocardial revascularization surgery. Among the patients who developed infections, 753 (49.7%) participants died^(17)^. Another study that followed the progress of 17 patients undergoing myocardial revascularization 30 days after hospital discharge showed a 6.3% SSI rate and a 5.8% mortality rate^(18)^.

This study revealed that the variables of temperature, presence of devices, and expected critical surgical events were statistically significant, indicating that they contribute to the occurrence of SSI after myocardial revascularization. Patients with a body temperature outside the range of 36 to 36.5°C had a higher risk of SSI. A study aimed at testing the hypothesis that intraoperative warming reduces major perioperative complications analyzed 5013 patients from 12 locations in China and the Cleveland Clinic in the United States. The study showed that 2507 patients underwent body warming with an average intraoperative temperature of 37.1°C, while 2506 patients had an average temperature of 35.6°C. As an outcome, 246 (9.9%) patients in the first group and 239 (9.6%) in the second group experienced issues such as myocardial injury, infections, cardiac arrest, or mortality^(19)^.

An international study aimed to demonstrate the relationship between perioperative catheter use and the incidence of SSI after adjusting for patient age, comorbidity severity, surgical approach, and instrument use. The study analyzed 39,893 patients 30 days after surgery and found that 1.6% of patients had SSI. Among these patients, 1.5% did not use a central venous catheter and had infections, while 5.8% used the catheter and developed post-surgical infections^(20)^.

A study conducted with the aim of determining the incidence and risk factors of SSI in hospitalized patients undergoing thoracic surgical procedures at a tertiary university hospital in Belgrade, Serbia, analyzed 3370 thoracic surgical procedures, and 205 (6.1%) of them had postoperative surgical infections. Among patients who had SSI, the use of invasive devices was also described as a risk factor associated with the occurrence of SSI. Additionally, 89 (43.4%) of patients who had SSI used central venous catheters, and 199 (97.1%) used postoperative drains^(21)^.

Expected critical events during the surgical procedure, such as bleeding, which increases the duration of surgery and hospitalization, were related to SSI in this study. A retrospective study conducted in Rio Grande do Sul aimed to characterize the clinical profile of patients undergoing cardiac surgery in the perioperative period and describe the follow-up after 30 days of hospital discharge. The analysis included 54 patients who underwent cardiac surgery, with 17 specifically undergoing myocardial revascularization, and it was found that two (11.8%) of these patients had postoperative bleeding within 48 hours^(18)^.

## Study limitations

However, it is important to highlight the limitations inherent in this study. One of the main limitations relates to the lack of detailed information about the surgical anesthetic procedure in the medical records. To mitigate these limitations, rigorous efforts were made in the analysis of available data.

## Contributions to the Nursing, Health, or Public Policy Field

This research contributes to building knowledge about the clinical practice of nurses in safe and high-quality care for surgical patients, as it highlights the factors that favor the occurrence of SSIs. Investments in future research are necessary for healthcare professionals to be aware of the evidence and how to use it to prevent complications resulting from surgical procedures, thus strengthening healthcare outcomes and patient safety.

## CONCLUSIONS

This study highlighted the predominance of elderly men, often with comorbidities such as hypertension and diabetes, classified as ASA 3. Regarding SSIs, approximately one-third of the participants developed this complication after undergoing cardiac surgery. Of these complications, a significant portion, equivalent to about one-tenth, resulted in death. We identified that factors such as body temperature outside the normothermic range, the presence of invasive devices, and the anticipation of critical events during surgical procedures were significant determinants of surgical site infections following myocardial revascularization. The total adherence score to the surgical checklist showed no relationship with SSI.

As these are preventable complications, it is emphasized that the perioperative team, especially nursing professionals, needs to be involved in care improvement processes through continuous education and access to the applicability of research findings in clinical practice.
